# Prediction of Antimicrobial Potential of a Chemically Modified Peptide From Its Tertiary Structure

**DOI:** 10.3389/fmicb.2018.02551

**Published:** 2018-10-26

**Authors:** Piyush Agrawal, Gajendra P. S. Raghava

**Affiliations:** ^1^CSIR-Institute of Microbial Technology, Chandigarh, India; ^2^Center for Computational Biology, Indraprastha Institute of Information Technology, Delhi, New Delhi, India

**Keywords:** chemically modified peptides, antimicrobial peptide prediction, machine learning technique, resistance, fingerprints, peptide therapeutics

## Abstract

Designing novel antimicrobial peptides is a hot area of research in the field of therapeutics especially after the emergence of resistant strains against the conventional antibiotics. In the past number of *in silico* methods have been developed for predicting the antimicrobial property of the peptide containing natural residues. This study describes models developed for predicting the antimicrobial property of a chemically modified peptide. Our models have been trained, tested and evaluated on a dataset that contains 948 antimicrobial and 931 non-antimicrobial peptides, containing chemically modified and natural residues. Firstly, the tertiary structure of all peptides has been predicted using software PEPstrMOD. Structure analysis indicates that certain type of modifications enhance the antimicrobial property of peptides. Secondly, a wide range of features was computed from the structure of these peptides using software PaDEL. Finally, models were developed for predicting the antimicrobial potential of chemically modified peptides using a wide range of structural features of these peptides. Our best model based on support vector machine achieve maximum MCC of 0.84 with an accuracy of 91.62% on training dataset and MCC of 0.80 with an accuracy of 89.89% on validation dataset. To assist the scientific community, we have developed a web server called “AntiMPmod” which predicts the antimicrobial property of the chemically modified peptide. The web server is present at the following link (http://webs.iiitd.edu.in/raghava/antimpmod/).

## Introduction

The emergence of drug-resistant pathogenic strains is one of the major threats for the survival of humans and livestock; antibiotics designed to eliminate these pathogens are losing their sensitivity (Price et al., 2012; Veltri et al., 2018). The rapid emergence of the antibiotic resistance has endangered the efficacy of antibiotics, and one of the potential causes of this is the misuse and overuse of antibiotics (Gould and Bal, 2013; Sengupta et al., 2013; Wright, 2014). Hence, there is a need to develop more potent and effective drugs to combat deadly diseases occurring worldwide. In the past few decades, peptide-based therapeutics has been preferred for the drug development over the small molecule-based drugs. Peptide-based drugs are highly selective, efficacious, safer and well tolerated compared to conventional small molecule-based drugs (Otvos and Wade, 2014). Proteins and peptide-based drugs cover around 10% of the pharmaceutical market as per the current report and will continue to grow in future (Bruno et al., 2013; Craik et al., 2013). Currently, more than 239 therapeutic proteins and peptides have been already approved by US-FDA (Fosgerau and Hoffmann, 2015; Usmani et al., 2017) and therefore researchers nowadays are focusing more on peptide-based drugs.

Broadly, peptides can be classified in four classes based on their therapeutic potential; (i) peptides as drug delivery vehicle, (ii) peptides as vaccine candidates, (iii) peptide-based inhibitors, and (iv) peptides-based disease biomarkers. Group I peptide can be used for delivering small molecules or drugs at their targets such as cell penetrating peptides, tumor homing peptides, brain barrier penetrating peptides (Gautam et al., 2012, 2013, 2016; Kapoor et al., 2012; Van Dorpe et al., 2012; Holton et al., 2013; Sharma et al., 2013; Agrawal et al., 2016; Wei et al., 2017; Wolfe et al., 2018). Group II peptides can be used for designing epitope-based vaccines or subunit vaccine; these are generally synthetic peptides or subunits of the whole organism commonly known as epitopes (Singh and Raghava, 2001; Ansari and Raghava, 2010; Singh et al., 2013; Shi et al., 2015; Oyarzún and Kobe, 2016; Alonso-Padilla et al., 2017; Jespersen et al., 2017). Group II peptides are one of the important categories of peptide-based therapeutics and can be clearly seen by the number of *in silico* methods developed in last decade (Rammensee et al., 1999; Singh and Raghava, 2003; Bhasin and Raghava, 2007; Zhang et al., 2008, 2011; Kringelum et al., 2012; Dhanda et al., 2013; Singh et al., 2013; Jurtz et al., 2017). These peptides generate memory cells and hence are very important nowadays for treating pathogenic infections. Epitopes/peptides are poor immunogens on their own and hence need the assistance of molecules known as adjuvants for increasing its potency (Sayers et al., 2012; Chaudhary et al., 2016; Nagpal et al., 2015, 2017, 2018).

Group III, peptides are inhibitors which can be used as drug molecules or inhibiting activity of drug targets (Eldar-Finkelman and Eisenstein, 2009; Groner et al., 2012; Beekman and Howell, 2016). These peptides kill pathogens by disrupting their cell membranes, by inhibiting their regulatory enzymes or by carrying out lysis (Ivanciuc et al., 2003; Rashid et al., 2009; Pirtskhalava et al., 2016; Wang et al., 2016, 2018; Singh et al., 2018). AMPs represent one of the broadest class of this group, for which number of databases and prediction methods have been developed in order to identify novel peptides which could act as drugs (Saha and Raghava, 2006; Gautam et al., 2014; Mehta et al., 2014; Kumar et al., 2015; Meher et al., 2017; Agrawal et al., 2018). Lastly, Group IV consists of those peptides which could potentially act as a biomarker and can be useful in developing different diagnostic kits (Shao, 2015; Bhalla et al., 2017). For example, peptides obtained from urine have been used as potential biomarkers for identifying multiple diseases (Siwy et al., 2011). Likewise, many computational methods have been created to maintain information related to peptides which could act as biomarkers (Zhang et al., 2006; Bhalla et al., 2017). Despite tremendous potential of peptides, there are many challenges in designing therapeutic peptides that include short half-life, challenges in oral delivery, immunotoxicity, cytotoxicity, etc. To address these issues, a number of computational resources has been developed in last two decades (Gupta et al., 2013; Sharma et al., 2014; Mathur et al., 2016, 2018; Liu et al., 2017; Porto et al., 2017b).

In the past few years, numerous methods have been developed to predict AMPs. Broadly, these methods can be classified in the following two groups (i) General methods and (ii) Class specific methods. The first group includes methods like CAMPR3, APD, AmPEP, and CS-AMPPred which predicts whether the given peptide is AMP or non-AMP (Porto et al., 2012; Wang, 2015; Waghu et al., 2016; Bhadra et al., 2018). CAMPR3 implements four different machine learning techniques for developing a prediction model (Waghu et al., 2016). APD is a physicochemical property based method that predicts AMP from the physicochemical property of the peptide (Wang, 2015). AmPEP is a random forest-based model developed using distribution patterns of amino acid properties along the sequence (Bhadra et al., 2018). CS-AMPPred is a support vector machine (SVM) based AMP prediction method developed for cysteine-stabilized peptides (Porto et al., 2012). The second group, i.e., class specific methods are those methods which are designed to predict peptides that can kill/inhibit specific class of organism and not in general. For example we have methods which predicts and designed peptides which are effective specifically either to bacteria or fungi or viruses or parasites. For example, Antibp and Antibp2 are two widely used SVM based methods developed to predict the antibacterial nature of the given peptide (Lata et al., 2007, 2010). AVPpred is developed for predicting antiviral peptide using machine learning technique SVM; features like the amino acid composition and physiochemical properties were used in this method (Thakur et al., 2012). Similarly, a method called Antifp has been developed for predicting antifungal peptide, it uses features like amino acid composition, binary profile (Agrawal et al., 2018). In addition, there are methods that predict the class of AMP (e.g., antibacterial, antifungal, and antiviral) like ClassAMP (Joseph et al., 2012). Similarly, another method iAMPpred predicts the probability of a peptide as an antibacterial, antifungal, and antiviral by providing the probability score for all the three classes (Meher et al., 2017). In past methods also developed for predicting AMPs in first step and class of AMP in the second step (Xiao et al., 2013).

Despite tremendous advances in the field of prediction of antimicrobial peptides, limited attempt has been made to predict antimicrobial peptides of chemically modified peptides. CS-AMPPred is a only method developed for predicting antimicrobial activity of a specific-type of chemical modification (cysteine-stabilized peptides). Best of our knowledge no method has been developed in past that can predict antimicrobial activity of a modified-peptide, which supports wide range of chemical modifications. In reality, most of the FDA approved therapeutic peptides are chemically modified, as the chemical modification is important for improving the stability of peptides in the body fluid, protection of peptide from the immune system, reducing the toxicity of peptide (Usmani et al., 2017; Al Musaimi et al., 2018). Thus it is need of time to develop a method that can predict antimicrobial inhibition potential of a chemically modified peptide from its tertiary structure. In this study, a systematic attempt had been made to predict AMP potential of a chemically modified peptide.

## Materials and Methods

### Dataset Creation

Modified AMPs were extracted from the SATPDB database (Singh et al., 2016) which maintains information about more than 19,000 natural and modified peptides. All those peptides which show any modification (terminus, chemical, and D-amino acids), is antimicrobial and whose tertiary structure is present were assigned as modified AMPs. In total, we got 948 such peptides. To develop any prediction method, we need negative dataset also. In our case, we selected those peptides as modified non-AMPs/negative dataset which exhibits any modifications (terminus, chemical, and D-amino acids), is non-antimicrobial in nature and whose tertiary structure is present in the SATPDB database. In the end, we got 931 such peptides. Therefore, we built the dataset of 948 positive peptides and 931 negative peptides.

### Internal and External Validation Dataset

The dataset was divided into two parts (i) training and (ii) validation dataset (Kumar et al., 2018). The training or main dataset consists of 80% of the total data, i.e., 758 modified AMPs and 745 non-AMPs. The validation dataset comprises of remaining 20% data, i.e., 190 modified AMPs and 186 non-AMPs. These peptides were selected randomly to avoid any biasness. Training dataset was used for internal validation, where models were trained and tested using fivefold cross-validation technique (Gautam et al., 2013). Performance of the best model achieved using training dataset was evaluated on the validation dataset, in the process commonly known as external validation.

### Additional Dataset

Discriminating between peptides which are compositionally similar but show different activity is a challenging task (Loose et al., 2006; Porto et al., 2017a). In order to evaluate the performance of different models developed in this study, we prepared another dataset “Mod_AMP_similar” having compositionally similar modified AMPs and non-AMPs. The positive set consists of those peptides which are present in the validation dataset whereas negative set consists of those peptides which are compositionally similar to the positive peptides. Compositionally similar peptides were identified by computing Euclidean distance between the diatomic composition of two peptides and the peptides having minimum Euclidean distance were selected. This kind of methodology has already been used in earlier studies (Kumar et al., 2008; Agrawal et al., 2018).

### Model Development

#### Feature Computation From Peptide Structures

##### Atom composition

Atom composition was calculated from modified AMPs and non-AMPs by converting peptides structures in SMILES format using openbabel (O’Boyle et al., 2011). The SMILES were further used to calculate atom composition of following atoms C, H, O, N, S, Cl, Br, and F. The atomic composition is calculated using formula 1 and provides a fixed length of eight vectors.

(1) $$\text{Fraction of atom (a)}=\frac{\text{total number of atom (a)}}{\text{total number of all possible atoms}}\times 100$$

where atom (a) is one out of all eight atoms.

##### Diatom composition

Diatom composition was computed in a similar manner as atom composition. The diatomic composition provides information about the pairs of atoms in each residue (e.g., C-C, C-O, C-N, etc.) of the peptides. The diatomic composition was computed using formula 2 which provided us a fixed length of 64 (8 × 8) vectors.

(2) $$\text{Fraction of atom (a)}=\frac{\text{total number of diatom (a)}}{\text{total number of all possible diatoms}}\times 100$$

where diatom (a) is one out of all 64 diatoms.

##### Chemical descriptors

Chemical or Molecular descriptors are terms that represents specific information of a given chemical molecule and determines its biological properties. Chemical descriptors represent the correlation between the physical, chemical and biological properties of a molecule and its chemical constitution in the form of numerical values (Roy et al., 2015). Majority of these chemical descriptors are classified on the basis of their dimensionality, which refers to the molecule representation from which descriptor values are calculated. Broadly, these descriptors are calculated as one dimensional (1D), two dimensional (2D), three dimensional (3D), and fingerprints (Xue and Bajorath, 2000). In the past, researchers have used the molecular descriptors to develop QSAR based prediction methods (Kumar et al., 2015). In our study, we used PaDEL software (Yap, 2011), which is a freely available software for calculating various descriptors of a given molecule. We calculated different types of descriptors which includes 2D descriptors and 10 different types of fingerprints. We performed feature selection technique to remove unnecessary descriptors, since all descriptors don’t correlate with the biological activity of the molecule, hence reducing noise from the dataset.

In this study, feature selection was performed using WEKA software (Data Mining: Practical Machine Learning Tools and Techniques, 2018) at default parameters. We selected “CfsSubsetEval” as an evaluator and “Best First” as a search method. The feature selection was performed in the forward direction with amount of backtracking, *N* = 5 and lookup size *D* = 1.

### Statistical Analysis

To find out the significant difference between modified AMPs and non-AMPs, we performed the Mann–Whitney–Wilcoxon test, which is a non-parametric test, using in-house R-script on the selected features of 2D descriptors, fingerprints, and combination of 2D descriptors and fingerprints.

### Binary Profiles

**FIGURE 1:**
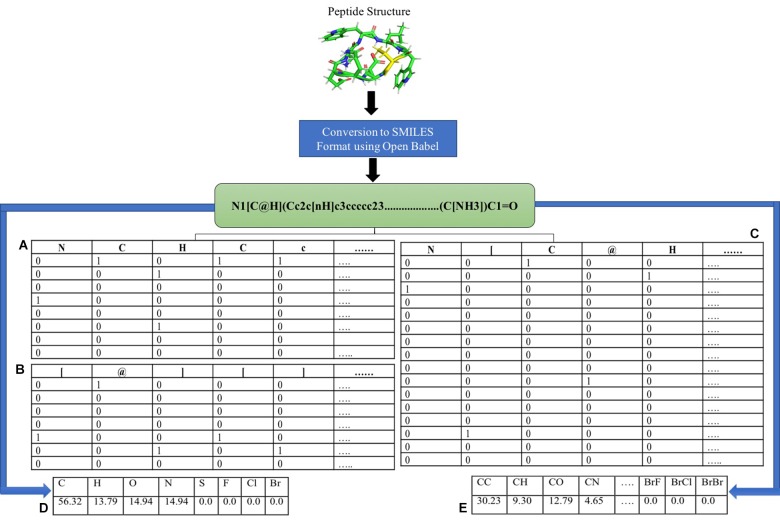
Feature extraction using SMILES format. Different features were calculated using SMILES format **(A)** binary profile generation of only atoms, **(B)** binary profile generation of only symbols, **(C)** binary profile generation of both symbol and atoms, **(D)** atom composition, and **(E)** diatom composition.

Differentiating AMPs with non-AMPs with similar peptide sequence is one of the challenging tasks. Although features like the composition and chemical descriptors can differentiate between AMPs and non-AMPs, they are unable to maintain the order of the residues in the peptide. To combat this situation, we converted the peptides into its SMILES format and extracted different numbers of atoms, symbols and both from the N and C terminus. Binary profiles of these atoms and symbols were generated, and prediction models were developed in three different categories. The first category includes only atoms present in the SMILES format, the second profile consists only of symbols, and the third contains the mixture of both. The binary profile was created from terminus (N, C, or both) for the first 25, 50, and 100 elements in case of only atoms and only symbols whereas for both (atom + symbol) first 50, 100, and 200 elements were considered. In the case of only atoms, there were total 8 atoms (C, H, O, N, S, F, Cl, and Br) where the presence of atom was represented by “1” and the absence by “0”, hence generating a vector of N × 8. In case of only symbols, we considered the most commonly occurring symbols (@, +, =, #, [,],.). These symbols are the chemical notations of a given chemical. For example, “-” is used to represent single bond, “=” is used to represent double bond, “#” is used to represent triple bond and so on. These symbols are represented in such a way so when given as an input, computer can easily understand it. Here also, the presence of symbol was indicated by “1” and the absence by “0”, hence leading to the vector of N × 7. In case of both, atom and symbols as mentioned above were taken, generating the vector of length N × 15. Binary profile generation is explained in Figure 1.

### Performance Measure

Performance of models were evaluated using different parameters which can be divided into two groups (i) threshold dependent parameters and (ii) threshold independent parameters.

The first group, i.e., threshold dependent parameters include Sensitivity (Sen), Specificity (Spc), Accuracy (Acc), and Matthew’s Correlation Coefficient (MCC). Here Sensitivity is defined as the true positive rate; Specificity is the true negative rate; Accuracy as the ability to differentiate between true positive and true negative whereas MCC is a correlation between observed and predicted value. These can be calculated using equations 3–6.

(3) $$\text{Sensitivity}=\frac{TP}{TP+FN}\times 100$$

(4) $$\text{Specificity}=\frac{TN}{TN+FP}\times 100$$

(5) $$\text{Accuracy}=\frac{TP+TN}{TP+FP+TN+FN}\times 100$$

(6) $$\text{Matthew’s Correlation Coefficient}=\frac{\left(TP*TN)-(FP*FN\right)}{\sqrt{\left(TP+FP)(TP+FN)(TN+FP)(TN+FN\right)}}\times 100$$

where TP and TN represents correctly predicted modified AMPs and non-AMPs, respectively. FP and FN represent wrongly predicted modified AMPs and non-AMPs, respectively.

The second group, i.e., threshold independent parameter includes AUROC, i.e., Area Under Receiver Operating Characteristic.

## Results

### Analysis

**FIGURE 2:**
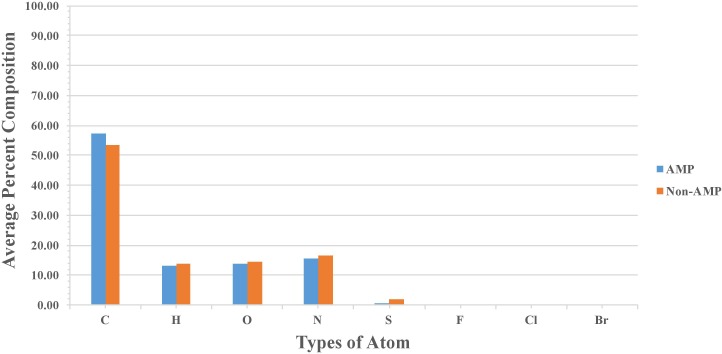
Comparison of atom composition present in modified AMPs and non-AMPs.

**FIGURE 3:**
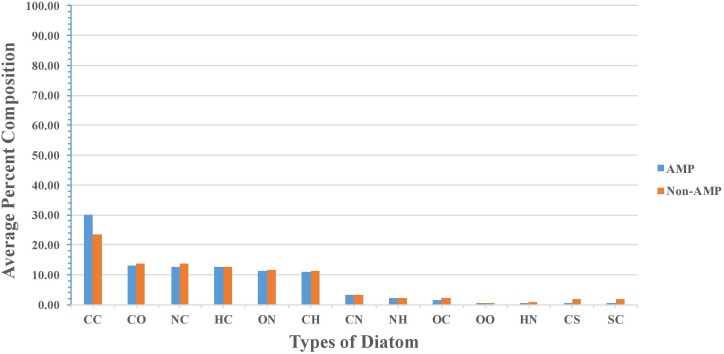
Comparison of diatom composition present in modified AMPs and non-AMPs.

Percent average composition of atoms present in modified AMPs and non-AMPs was computed for understanding the type of atom preference. Overall, the profile was found to be more or less the same in modified AMPs and non-AMPs. AMPs were found to be slightly higher in “C” atom compared to non-AMPs whereas non-AMPs were found to be higher in “S” atom compared to AMPs. Halogens were found to be absent in AMPs and non-AMPs (Figure 2). We also analyzed the diatoms composition and observe that diatom “CC” is dominant in AMPs whereas “NC,” “OC,” “CS,” and “SC” were more abundant in non-AMPs (Figure 3).

### Machine Learning Based Prediction Model

Peptide tertiary structure can present different types of chemical modifications. Therefore, the structure of peptides was utilized to compute the feature and predict its antimicrobial nature. Various machine learning techniques like SVM (Cortes and Vapnik, 1995), Random Forest, Naive Bayes, J48, and SMO were used to develop the prediction model in the study. These models utilize different features for discriminating modified AMPs form non-AMPs. The results are explained below in the following sections:

### Composition Based Prediction

We developed prediction models for the atomic and diatomic composition of the peptide using various classifiers. In case of atomic composition, SVM model performed better than other models with an accuracy of 86.83% with MCC of 0.74 on the training dataset and accuracy of 83.51% and MCC of 0.67 on the validation dataset (Table 1). For diatomic composition, Random Forest model achieved the highest accuracy of 89.75% with MCC of 0.80 on training dataset whereas on validation dataset the model showed the accuracy of 87.50% and MCC of 0.75 (Table 2).

**Table 1:** The performance of atom composition based models developed using different machine learning techniques.

Machine learning techniques (parameters)	Main dataset					Validation dataset				
	Sen	Spc	Acc	MCC	AUROC	Sen	Spc	Acc	MCC	AUROC
SVM (*g* = 0.1, *c* = 9, *j* = 1)	90.37	83.22	86.83	0.74	0.92	89.47	77.42	83.51	0.67	0.88
Random Forest (Ntree = 20)	89.58	81.74	85.70	0.72	0.93	91.05	81.72	86.44	0.73	0.90
SMO (*g* = 0.1, *c* = 3)	88.39	82.15	85.30	0.71	0.85	90.00	76.88	83.51	0.68	0.83
J48 (*c* = 0.15, *m* = 5)	88.13	80.27	84.23	0.69	0.88	85.79	77.96	81.91	0.64	0.85
Naive Bayes (Default)	89.84	62.55	76.31	0.55	0.77	87.89	60.75	74.47	0.51	0.79

*Sen, Sensitivity; Spc, Specificity; Acc, Accuracy; MCC, Matthew’s Correlation Coefficient; AUROC, Area Under the Receiver Operating Characteristic curve*.

**Table 2:** The performance of diatom composition based models developed using different machine learning techniques.

Machine learning techniques (parameters)	Main dataset					Validation dataset				
	Sen	Spc	Acc	MCC	AUROC	Sen	Spc	Acc	MCC	AUROC
SVM (*g* = 0.05, *c* = 15, *j* = 2)	89.71	86.85	88.29	0.77	0.93	90.53	81.72	86.17	0.73	0.92
Random Forest (Ntree = 150)	94.20	85.23	89.75	0.80	0.96	92.11	82.80	87.50	0.75	0.93
SMO (*g* = 0.1, *c* = 5)	88.79	87.92	88.36	0.77	0.88	88.95	83.33	86.17	0.72	0.86
J48 (*c* = 0.25, *m* = 1)	89.71	83.22	86.49	0.73	0.88	86.84	83.87	85.37	0.71	0.86
Naive Bayes (Default)	87.86	63.09	75.58	0.53	0.74	87.37	62.37	75.00	0.51	0.74

*Sen, Sensitivity; Spc, Specificity; Acc, Accuracy; MCC, Matthew’s Correlation Coefficient; AUROC, Area Under the Receiver Operating Characteristic curve*.

### Chemical Descriptors Based Prediction

Individual models were developed for 2D descriptors and fingerprints as well as the single model was developed combining features of 2D descriptors and fingerprints. These model were developed on the complete features as well as features obtained after feature selection process (see section “Materials and Methods”). In case of 2D descriptors, initially 231 descriptors were calculated, and SVM based model achieved the highest accuracy of 61.29% with MCC of 0.23 on training dataset and accuracy of 60.90% and MCC of 0.28 on validation dataset (Table 3). We applied feature selection process on these 231 features reducing them to 4. List of these features is provided in Supplementary Table S1. Machine learning techniques were applied on these selected features, and we observed that SVM based model achieved the highest accuracy of 80.68% with MCC of 0.62 on training dataset and accuracy of 79.79% and MCC of 0.60 on validation dataset (Table 3).

**Table 3:** The performance of 2D descriptors based models developed using different machine learning techniques.

Machine learning techniques (parameters)	Main dataset					Validation dataset				
	Sen	Spc	Acc	MCC	AUROC	Sen	Spc	Acc	MCC	AUROC
SVM full feature (*g* = 1e-05, *c* = 2, *j* = 2)	56.88	65.77	61.29	0.23	0.70	30.53	91.94	60.90	0.28	0.75
SVM after feature selection (*g* = 0.1, *c* = 1, *j* = 1)	84.92	76.38	80.68	0.62	0.85	84.74	74.73	79.79	0.60	0.87
Random Forest (Ntree = 20)	82.01	77.45	79.75	0.60	0.88	83.68	75.81	79.79	0.60	0.86
SMO (*g* = 0.1, *c* = 1)	87.04	74.63	80.88	0.62	0.81	85.79	71.51	78.72	0.58	0.79
J48 (*c* = 0.25, *m* = 6)	81.08	77.99	79.55	0.59	0.85	83.68	74.19	78.99	0.58	0.83
Naive Bayes (Default)	87.30	62.82	75.15	0.52	0.83	87.89	61.29	74.73	0.51	0.81

*Sen, Sensitivity; Spc, Specificity; Acc, Accuracy; MCC, Matthew’s Correlation Coefficient; AUROC, Area Under the Receiver Operating Characteristic curve*.

In case of fingerprints initially, we calculated 4812 features and developed the SVM model which shows 91.62% accuracy with 0.84 MCC on training dataset and 89.89% accuracy and 0.80 MCC on the validation dataset (Table 4). We applied feature selection technique on these features reducing them to a total of 18 features (Supplementary Table S2). The SVM based model developed on these 18 features showed the accuracy of 81.77% with MCC of 0.64 on the training dataset and accuracy of 79.26% and MCC of 0.59 on the validation dataset. Therefore, we developed different machine learning models using complete features and reported the performance in Table 4.

**Table 4:** The performance of fingerprints based models developed using different machine learning techniques.

Machine learning techniques (parameters)	Main dataset					Validation dataset				
	Sen	Spc	Acc	MCC	AUROC	Sen	Spc	Acc	MCC	AUROC
SVM full feature (*g* = 0.001, *c* = 4, *j* = 1)	95.91	87.25	91.62	0.84	0.97	93.16	86.56	89.89	0.80	0.97
SVM after feature selection (*g* = 0.1, *c* = 6, *j* = 1)	82.85	80.67	81.77	0.64	0.87	82.63	75.81	79.26	0.59	0.84
Random Forest (Ntree = 100)	92.88	90.07	91.48	0.83	0.98	92.63	89.25	90.96	0.82	0.97
SMO (*g* = 0.001, *c* = 4)	91.29	89.66	90.49	0.81	0.90	89.47	90.32	89.89	0.80	0.90
J48 (*c* = 0.4, *m* = 1)	90.50	88.99	89.75	0.80	0.88	88.95	86.02	87.50	0.75	0.85
Naive Bayes (Default)	84.30	64.56	74.52	0.50	0.74	78.42	65.05	71.81	0.44	0.72

*Sen, Sensitivity; Spc, Specificity; Acc, Accuracy; MCC, Matthew’s Correlation Coefficient; AUROC, Area Under the Receiver Operating Characteristic curve*.

In case of all combined features (2D descriptors + fingerprints), we calculated 5043 features initially. SVM model developed using complete feature showed the accuracy of 59.59% with MCC of 0.29 on training dataset and accuracy of 59.57% and MCC of 0.28 on the validation dataset. Feature selection technique reduced the number of features from 5043 to 20 (Supplementary Table S3). SVM model developed on these features showed the higher accuracy of 81.76% and MCC of 0.64 on the training dataset, and on the validation dataset, it achieved an accuracy of 82.71% and MCC of 0.65. Performance of other classifiers obtained on these features is provided in Supplementary Table S4. Random Forest performed best among all the models with accuracy of 90.35% and MCC of 0.81 on training dataset and accuracy of 88.56% and MCC of 0.77 on the validation dataset.

### Significance of Features

Significant difference was observed between the positive and negative features based on *p*-values. For most of the features, we found the *p*-value less than 0.05. Therefore, we can conclude that these features are important and can be used to discriminate between modified AMPs and non-AMPs. Mean value of features (positive and negative) along with their *p*-value for 2D descriptors, fingerprints and hybrid feature (2D descriptors + fingerprints) is provided in Supplementary Tables S1–S3, respectively.

### Binary Profile Based Prediction

In this part of the study, the binary profile was generated using SMILES format, and prediction models were developed in three different categories. In the first category, where only atoms were taken we developed SVM based models for the first 25, 50, and 100 elements from N terminus (N25, N50, and N100), C terminus (C25, C50, and C100) and joining both termini (N25C25, N50C50, and N100C100). We obtained the best performance for the N100C100 binary profile with an accuracy of 89.84% and MCC of 0.80 on training dataset and accuracy of 87.37% and MCC of 0.75 on validation dataset (Table 5). In the second category, we considered only symbols and calculated the binary profile in the same manner as for the first category. Here also, N100C100 binary profile achieved the highest accuracy of 87.42% and MCC of 0.75 on training dataset and accuracy of 80.53% and MCC of 0.61 on the validation dataset (Supplementary Table S5). For the last category, where both symbol and atoms were considered we calculated the binary profile for the first 50, 100, and 200 elements from N-terminus, C-terminus, and by joining elements of both termini. Here, the model developed on N200C200 binary profile performed better than other models with an accuracy of 89.35% and MCC of 0.79 on training dataset and accuracy of 85.86% and MCC of 0.72 on validation dataset (Table 6).

**Table 5:** The performance of SVM based models developed using binary profile of atoms obtained from terminals of SMILES format.

Feature (parameters)	Main dataset					Validation dataset				
	Sen	Spc	Acc	MCC	AUROC	Sen	Spc	Acc	MCC	AUROC
N25 (*g* = 0.05, *c* = 8, *j* = 2)	77.63	75.68	76.67	0.53	0.83	79.59	85.71	82.42	0.65	0.91
N50 (*g* = 0.01, *c* = 3, *j* = 3)	83.17	79.31	81.27	0.63	0.88	90.58	86.40	88.59	0.77	0.93
N100 (*g* = 0.005, *c* = 6, *j* = 2)	85.71	84.18	84.90	0.70	0.93	85.04	84.93	84.98	0.70	0.93
C25 (*g* = 0.01, *c* = 5, *j* = 4)	79.11	70.43	74.70	0.50	0.79	89.19	74.51	82.16	0.65	0.83
C50 (*g* = 0.1, *c* = 1, *j* = 1)	83.47	72.08	77.94	0.56	0.85	88.31	74.83	81.73	0.64	0.91
C100 (*g* = 0.001, *c* = 3, *j* = 2)	82.97	81.85	82.38	0.65	0.89	89.55	77.55	83.27	0.67	0.92
N25C25 (*g* = 0.01, *c* = 5, *j* = 2)	85.69	84.82	85.27	0.71	0.91	84.71	82.31	83.55	0.67	0.92
N50C50 (*g* = 0.05, *c* = 2, *j* = 1)	89.79	87.16	88.47	0.77	0.95	87.43	85.63	86.53	0.73	0.95
N100C100 (*g* = 0.01, *c* = 6, *j* = 1)	90.15	89.58	89.84	0.80	0.96	90.51	84.62	87.37	0.75	0.96

*Sen, Sensitivity; Spc, Specificity; Acc, Accuracy; MCC, Matthew’s Correlation Coefficient; AUROC, Area Under the Receiver Operating Characteristic curve; N25/N50/N100, first 25/50/100 elements from N-terminal; C25/C50/C100, first 25/50/100 elements from C-terminal; N25C25/N50C50/N100C100, first 25/50/100 elements from N-terminal as well as from C-terminal joined together*.

**Table 6:** The performance of SVM based models developed using binary profile of atoms and symbols together obtained from terminals of SMILES format.

Feature (parameters)	Main dataset					Validation dataset				
	Sen	Spc	Acc	MCC	AUROC	Sen	Spc	Acc	MCC	AUROC
N50 (*g* = 0.005, *c* = 6, *j* = 2)	75.50	75.76	75.62	0.51	0.80	63.21	92.63	77.11	0.58	0.89
N100 (*g* = 0.01, *c* = 2, *j* = 3)	81.26	80.39	80.84	0.62	0.88	77.62	78.79	78.18	0.56	0.89
N200 (*g* = 0.01, *c* = 1, *j* = 2)	85.28	81.57	83.32	0.67	0.92	81.06	77.48	79.15	0.58	0.90
C50 (*g* = 0.01, *c* = 5, *j* = 2)	72.47	72.13	72.30	0.45	0.79	78.10	71.43	74.88	0.50	0.84
C100 (*g* = 0.01, *c* = 3, *j* = 1)	77.93	75.83	76.94	0.54	0.83	84.42	78.72	81.69	0.63	0.89
C200 (*g* = 0.005, *c* = 5, *j* = 1)	80.80	79.66	80.20	0.60	0.89	83.09	82.05	82.53	0.65	0.92
N50C50 (*g* = 0.005, *c* = 8, *j* = 3)	86.45	84.19	85.36	0.71	0.91	83.97	87.84	85.86	0.72	0.92
N100C100 (*g* = 0.01, *c* = 2, *j* = 1)	90.38	86.25	88.35	0.77	0.96	86.90	84.94	85.93	0.72	0.94
N200C200 (*g* = 0.005, *c* = 1, *j* = 2)	91.59	87.46	89.35	0.79	0.96	89.29	82.93	85.86	0.72	0.94

*Sen, Sensitivity; Spc, Specificity; Acc, Accuracy; MCC, Matthew’s Correlation Coefficient; AUROC, Area Under the Receiver Operating Characteristic curve; N50/N100/N200, first 50/100/200 elements from N-terminal; C50/C100/C200, first 50/100/200 elements from C-terminal; N50C50/N100C100/N200C200, first 50/100/200 elements from N-terminal as well as from C-terminal joined together*.

### Additional Dataset Performance

We evaluated the performance of the model developed on the additional dataset termed as “Mod_AMP_similar”. Performance of the SVM models developed on different features like the composition, chemical descriptors and binary profiles is compared in Table 7. It can be clearly seen that model developed using fingerprints with an accuracy of 90.26% and MCC of 0.81 followed by the model developed using N100C100 binary profile where only atoms were considered performed best with an accuracy of 89.66% and MCC of 0.80.

**Table 7:** The performance of SVM based models developed using different features on additional dataset.

Features (parameters)	Mod_AMP_similar Dataset				
	**Sen**	**Spc**	**Acc**	**MCC**	**AUROC**
Atom composition (*g* = 0.1, *c* = 9, *j* = 1)	89.47	43.68	66.58	0.37	0.77
Diatom composition (*g* = 0.05, *c* = 15, *j* = 2)	88.42	71.58	80.00	0.61	0.88
2D descriptors (*g* = 0.1, *c* = 1, *j* = 1)	84.74	32.63	58.68	0.20	0.66
Fingerprints (*g* = 0.001, *c* = 4, *j* = 1)	93.16	87.37	90.26	0.81	0.97
Hybrid features (2D + fingerprints) (*g* = 0.005, *c* = 7, *j* = 2)	84.74	58.95	71.84	0.45	0.81
N100C100 Binary profile (only atoms) (*g* = 0.01, *c* = 6, *j* = 1)	90.51	89.44	89.66	0.80	0.97
N100C100 Binary profile (only symbols) (*g* = 0.005, *c* = 7, *j* = 2)	76.98	91.10	84.21	0.60	0.94
N200C200 Binary profile (atom + symbols) (*g* = 0.005, *c* = 1, *j* = 2)	89.29	89.12	89.20	0.78	0.96

*Sen, Sensitivity; Spc, Specificity; Acc, Accuracy; MCC, Matthew’s Correlation Coefficient; AUROC, Area Under the Receiver Operating Characteristic curve; N100C100, first 100 elements form N-terminus and C-terminus, respectively; N200C200, first 200 elements form N-terminus and C-terminus, respectively*.

### Implementation of the Web Server

To assist the researchers, we have developed a web server named “AntiMPmod” where the best prediction model has been incorporated. The PREDICTION module takes a tertiary structure of the modified peptide (PDB format) as an input for performing prediction. If a user does not have its own modified peptide tertiary structure, user can generate the peptide tertiary structure up to 25 residues in length using the server “PEPstrMOD”^1^ (Singh et al., 2015). This server was developed by our group specifically for tertiary structure prediction of the modified peptide. A user can select the desired modification from the wide variety of modification options present at the server. Once the structure is generated, the user can submit the structure in the PREDICTION module and can predict whether the provided peptide was AMP or non-AMP. Another module termed as “DOWNLOAD”, provides the dataset used in this study.

### Standalone

In order to assist the researchers, we have also developed the standalone software of AntiMPmod. User needs to pull the docker image “raghavagps/gpsraghava” and can run the software using the PERL code provided inside the folder termed as “gpsr.”

## Discussion

Rapidly growing resistance and failure of conventional antibiotics to treat pathogenic infections are one of the serious public health concerns (Komolafe, 2003; Fair and Tor, 2014). In the “post-antibiotic era”, researchers are heading toward the peptide-based antibiotics due to its various advantages over the antibiotics. Natural AMPs because of its various therapeutic properties (bactericidal property, immunomodulatory activity, a broad spectrum of activity, etc.) have rapidly captured attention as novel drug candidates. AMPs are short innate immunity peptides present in almost all living organism and act as a universal host defense molecule. AMPs belong to diverse families which include cathelicidins (Zanetti, 2005), defensins (Lehrer, 2004), cercopins (Boman, 2000), and magainins (Berkowitz et al., 1990). AMPs possess a broad range of properties in terms of their physiochemical properties, composition, 3D structure and mechanism of action. Majority of them are small, positively charged and amphipathic, 4–100 amino acid in length with diverse amino acid composition (Gentilucci et al., 2006; Wang, 2012). Recently, the 3D structure of the natural AMPs has been classified into four broad families (i) α-helical (possess helix), (ii) β-sheet (consists of sheet usually stabilized by disulfide bonds), (iii) αβ (consists of both helix and sheet), and (iv) non-αβ (do not have clearly defined structures) (Fjell et al., 2012). AMPs mostly kill their targets by various mechanisms such as cell membrane damage or pore formation that leads to efflux of nutrients and ions (Melo et al., 2009), DNA interference or signaling responses (Wimley and Hristova, 2011).

Natural ecosystem has been proven a reservoir of a wide variety of compounds that may be explored for the development of potential drug molecule. Researchers have explored several biomes and discovered a large number of AMPs from the microorganism, plants and animals having therapeutic potentials, for example, bovine lactoferrin, LL-37 (de Castro and Franco, 2015; Mahlapuu et al., 2016). Literature is full of such discoveries, and a large number of databases have been developed which maintains a wide variety of information of AMPs (Novković et al., 2012; Pirtskhalava et al., 2016; Waghu et al., 2016; Wang et al., 2016). However, most of the natural AMPs based drug have not reached clinical trials. This is largely due to the high structural complexity of the compound, low compound stability, low activity toward the target, compound side effects, degradation of the compound by the host enzyme, and the high drug development cost (de Castro and Franco, 2015). To overcome the above-mentioned problems, researchers have tried to design the modified compounds by incorporating various chemical modifications such as capping, halogenation, hydroxylation, glycosylation, phosphorylation, designing antimicrobial peptide mimetic, AMP congeners, AMP conjugates, and immobilized AMPs. Details of the different kind of modifications for the novel antimicrobial peptide engineering are reviewed by Wang (2012). Computational methods have shown a wide variety of success in the field of drug discovery process (Dhanda et al., 2017).

**FIGURE 4:**
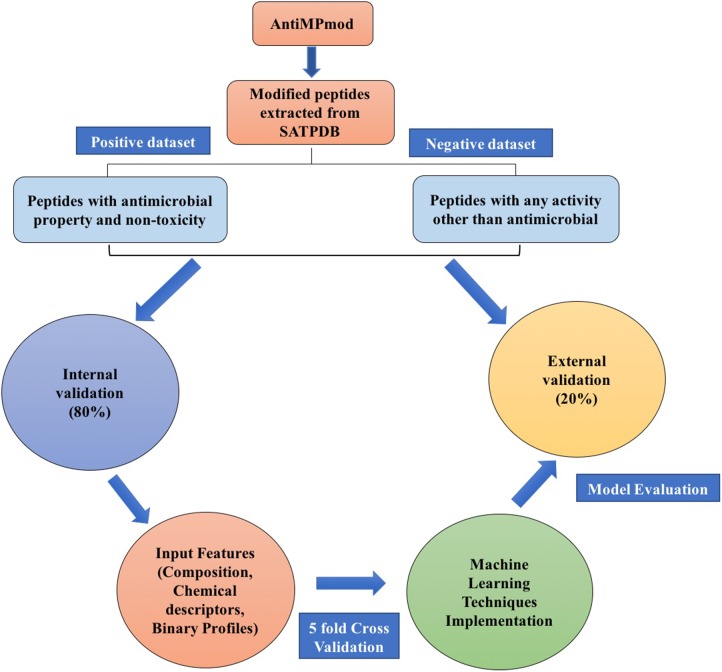
Schematic representation of AntiMPmod workflow.

**FIGURE 5:**
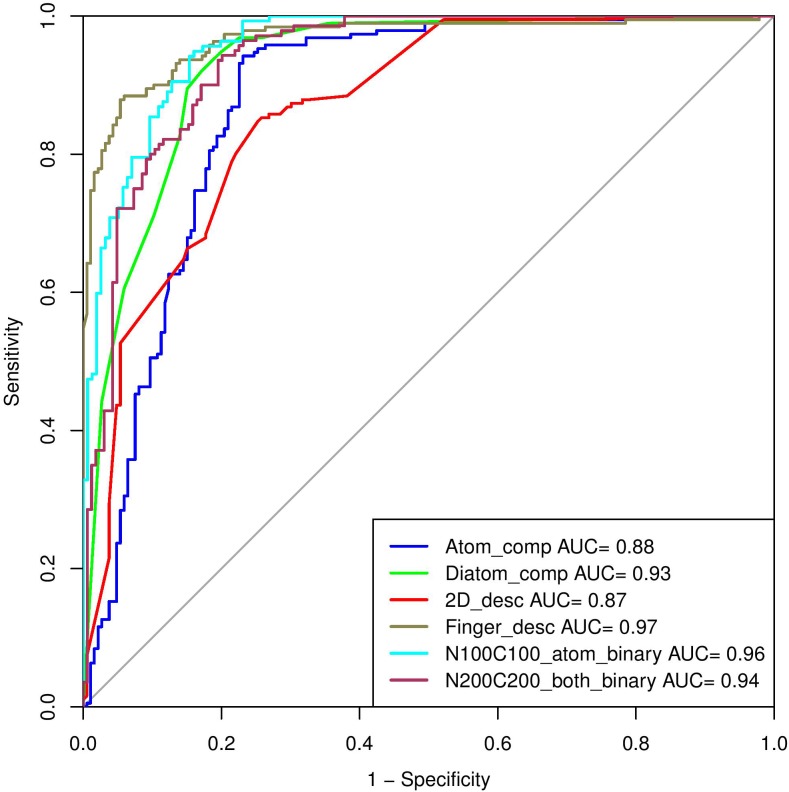
The performance of best models on independent dataset, in terms of ROC curves developed using different input features.

In the past, numerous methods have been made for predicting and designing novel AMP, but one of the biggest limitations of these methods is that they can only handle the peptide sequence containing natural residues. In the current study, we have developed a prediction method which predicts the antimicrobial property of a given chemically modified peptide using its tertiary structure. One of the major advantage of using 3D structure over sequence is the inclusion of chemical modification information during prediction which is nearly impossible with sequence based prediction. It is because representing chemical modification in a sequence is a challenging task. Also, molecular descriptors can be calculated easily using 3D structure which covers information of all the chemical properties of a modified peptide in comparison to sequence. These structure-based methods have their own limitations which includes requirement of tertiary structure of peptides. Experimental techniques (e.g., X-ray crystallography, NMR, and cryo-electron microscopy) for determination of peptide structure are time consuming and costly. Computational techniques like molecular dynamics and method like PEPstrMOD for predicting structure from sequence have their own limitations including accuracy and speed of prediction. The overall scheme of the AntiMPmod has been shown in Figure 4. We extracted the modified AMPs and non-AMPs from the SATPDB database and analyzed these structures. We found different kind of modifications such as acetylation, amidation, methylation, glycosylation, and presence of non-natural residues such as ornithine, norleucine, D amino acids, etc. Secondary structure content was analyzed by running DSSP (Kabsch and Sander, 1983; Joosten et al., 2011) and we found that modified AMPs were highly dominated by turns, coils and extended loop regions (∼62.5%) followed by helical content (∼36%) and very little amount of sheet content (∼1.5%). We extracted different kind of features such as composition, chemical descriptors, fingerprints and binary profiles from these modified peptides and used them for developing prediction models using various machine learning classifiers. We found that SVM based model utilizing fingerprints as feature performed best among all the models followed by the model developed using binary profiles. In case of binary profile based models, we observed as the number of terminus elements was increasing their performance too was increasing and when we join the elements of both termini, they performed better than their individual terminus. This suggests that terminus information plays a significant role in predicting the nature of peptide. In addition to this, we created an additional dataset where positive and negative peptides were compositionally similar. We evaluated the performance of different models on this dataset and found that binary profile model which considers only atoms and fingerprint-based model performed best and can classify the modified AMPs and non-AMPs with higher accuracy. Overall summary of the result of this study is given in Table 8 where we have mentioned the best performance obtained by the prediction model on different input features. Performance achieved on the independent dataset by the best models developed using various input features is shown in the Figure 5, where we have calculated AUROC. We implemented our best model in the web server “AntiMPmod” and believes that this study will be helpful for the researchers working in the field of drug discovery.

**Table 8:** The performance of best models developed using different machine learning techniques based on different features.

Feature (machine learning technique with parameters)	Main dataset					Validation dataset			
	
	Sen	Spc	Acc	MCC	AUROC	Sen	Spc	Acc	MCC
Atom composition (SVM, *g* = 0.1, *c* = 9, *j* = 1)	90.37	83.22	86.83	0.74	0.92	89.47	77.42	83.51	0.67
Diatom composition (Random Forest, Ntree = 150)	94.20	85.23	89.75	0.80	0.96	92.11	82.80	87.50	0.75
2D descriptors (SVM, *g* = 0.1, *c* = 1, *j* = 1)	84.92	76.38	80.68	0.62	0.85	84.74	74.73	79.79	0.60
Fingerprints (SVM, *g* = 0.001, *c* = 4, *j* = 1)	95.91	87.25	91.62	0.84	0.97	93.16	86.56	89.89	0.80
N100C100 Binary profile (only atoms) (SVM, *g* = 0.01, *c* = 6, *j* = 1)	90.15	89.58	89.84	0.80	0.96	90.51	84.62	87.37	0.75
N200C200 Binary profile (atoms + symbols both) (SVM, *g* = 0.005, *c* = 1, *j* = 2)	91.59	87.46	89.35	0.79	0.96	89.29	82.93	85.86	0.72

*Sen, Sensitivity; Spc, Specificity; Acc, Accuracy; MCC, Matthew’s Correlation Coefficient; AUROC, Area Under the Receiver Operating Characteristic curve*.

## Author Contributions

PA collected the data, created the datasets and the back-end server, performed all the experiments, and developed the front end user interface. PA and GPSR analyzed the results and wrote the manuscript. GPSR conceived the idea and coordinated the project.

## Conflict of Interest Statement

The authors declare that the research was conducted in the absence of any commercial or financial relationships that could be construed as a potential conflict of interest.

## Footnotes

1 http://webs.iiitd.edu.in/raghava/pepstrmod/
