# Supplementary material for: Prediction of Antimicrobial Potential of a Chemically Modified Peptide From Its Tertiary Structure
Source: Front Microbiol. 2018 Oct 26;9:2551. doi: 10.3389/fmicb.2018.02551 (PMC6212470; doi:10.3389/fmicb.2018.02551)
Supplement: Supplementary file 1 [file Table_1.DOCX]

**Prediction of Antimicrobial Potential of a Chemically Modified Peptide from its Tertiary Structure**

**Piyush Agrawal^1,2^ and Gajendra P.S. Raghava*^2^**

1. CSIR-Institute of Microbial Technology, Chandigarh, India.

2. Center for Computational Biology, Indraprastha Institute of Information Technology, New Delhi, India.

**Emails of Authors:**

PA: [piyush_11@imtech.res.in](mailto:piyush_11@imtech.res.in)

**^*^ Corresponding author**

Professor, Center for Computational Biology, Indraprastha Institute of Information Technology,

Okhla Industrial Estate, Phase III, New Delhi 110020,

India. Tel.: +91 011 26907444

E-mail address: [raghava@iiitd.ac.in](mailto:raghava@iiitd.ac.in)

**Supplementary data**

**Table S1. List of 2D features with their positive mean value, negative mean value and p-value.**

| **Sr. No.** | **Feature** | **Feature Description** | **Mean Pos Value** | **Mean Neg Value** | **Mean Difference (Pos-Neg)** | **W value*** | **P-value** |
| --- | --- | --- | --- | --- | --- | --- | --- |
| 1 | nC | Number of Cysteine | 0.21 | 2.88 | -2.67 | 190991 | 0.00 |
| 2 | nV | Number of Valine | 1.17 | 1.56 | -0.39 | 594542.5 | 0.00 |
| 3 | nS | Number of Sulphur atoms | 4.61 | 2.25 | 2.36 | 203522.5 | 0.00 |
| 4 | khs.sSH | Type of Kappa Shape Indices descriptor | 0.17 | 2.59 | -2.42 | 217935.5 | 0.00 |

* W (Wilcoxon test statistic) value is sum of the ranks in one of both groups.

**Table S2. List of fingerprints features with their positive mean value, negative mean value and p-value.**

| **Sr. No.** | **Feature** | **Feature Description** | **Mean Pos Value** | **Mean Neg Value** | **Mean Difference (Pos-Neg)** | **W value*** | **P-value** |
| --- | --- | --- | --- | --- | --- | --- | --- |
| 1 | FP339 | Fingerprint of length 1024 and search depth of 8 | 0.06 | 0.02 | 0.04 | 458330.5 | 0.00 |
| 2 | FP344 | Fingerprint of length 1024 and search depth of 8 | 0.90 | 0.98 | -0.08 | 403250.5 | 0.00 |
| 3 | FP396 | Fingerprint of length 1024 and search depth of 8 | 0.01 | 0.15 | -0.14 | 377735.5 | 0.00 |
| 4 | ExtFP664 | Extend the fingerprinter with additional bits describing ring features | 0.01 | 0.43 | -0.42 | 256383 | 0.00 |
| 5 | ExtFP824 | Extend the fingerprinter with additional bits describing ring features | 0.00 | 0.16 | -0.16 | 372547 | 0.00 |
| 6 | ExtFP830 | Extend the fingerprinter with additional bits describing ring features | 0.00 | 0.51 | -0.51 | 218006 | 0.00 |
| 7 | ExtFP848 | Extend the fingerprinter with additional bits describing ring features | 0.04 | 0.20 | -0.16 | 368009 | 0.00 |
| 8 | ExtFP994 | Extend the fingerprinter with additional bits describing ring features | 0.02 | 0.58 | -0.56 | 195583.5 | 0.00 |
| 9 | GraphFP41 | Specialized version of the Fingerprinter which does not take bond orders into account | 0.02 | 0.32 | -0.30 | 310308.5 | 0.00 |
| 10 | GraphFP226 | Specialized version of the Fingerprinter which does not take bond orders into account | 0.19 | 0.10 | 0.09 | 478649 | 0.00 |
| 11 | GraphFP282 | Specialized version of the Fingerprinter which does not take bond orders into account | 0.01 | 0.56 | -0.55 | 194763 | 0.00 |
| 12 | GraphFP292 | Specialized version of the Fingerprinter which does not take bond orders into account | 0.00 | 0.51 | -0.51 | 218006 | 0.00 |
| 13 | GraphFP714 | Specialized version of the Fingerprinter which does not take bond orders into account | 0.00 | 0.19 | -0.19 | 357387.5 | 0.00 |
| 14 | GraphFP1011 | Specialized version of the Fingerprinter which does not take bond orders into account | 0.00 | 0.43 | -0.43 | 254978 | 0.00 |
| 15 | GraphFP1023 | Specialized version of the Fingerprinter which does not take bond orders into account | 0.01 | 0.57 | -0.56 | 195194.5 | 0.00 |
| 16 | MACCSFP114 | MACCS keys | 0.62 | 0.39 | 0.23 | 542980 | 0.00 |
| 17 | PubchemFP35 | >= 4 S | 0.02 | 0.58 | -0.56 | 192308 | 1.00 |
| 18 | SubFPC36 | Presence of SMARTS Patterns for Functional Group Classification by Christian Laggner | 0.17 | 2.59 | -2.42 | 218341 | 0.00 |

* W (Wilcoxon test statistic) value is sum of the ranks in one of both groups.

**Table S3. List of hybrid (2D+fingerprints) features with their positive mean value, negative mean value and p-value.**

| **Sr. No.** | **Features** | **Feature Description** | **Mean Pos Value** | **Mean Pos Value** | **Mean Difference (Pos-Neg)** | **W value*** | **P-value** |
| --- | --- | --- | --- | --- | --- | --- | --- |
| 1 | nI | Number of Isoleucine | 1.17 | 0.53 | 0.64 | 577852 | 0.00 |
| 2 | nV | Number of Valine | 4.63 | 2.25 | 2.38 | 595528.5 | 0.00 |
| 3 | nS | Number of Sulphur atom | 0.42 | 3.02 | -2.60 | 204293.5 | 0.00 |
| 4 | ATSc3 | Autocorrelation Descriptor | -0.32 | -0.35 | -0.67 | NA | 0.00 |
| 5 | khs.sSH | Type of Kappa Shape Indices descriptor | 0.17 | 2.59 | -2.42 | 218704.5 | 0.00 |
| 6 | TopoPSA | Topological Polar Surface Area | 638.57 | 830.35 | -191.78 | NA | 0.00 |
| 7 | ExtFP664 | Extend the fingerprinter with additional bits describing ring features | 0.01 | 0.43 | -0.42 | 256115.5 | 0.00 |
| 8 | ExtFP830 | Extend the fingerprinter with additional bits describing ring features | 0.00 | 0.51 | -0.51 | 217778 | 0.00 |
| 9 | ExtFP931 | Extend the fingerprinter with additional bits describing ring features | 0.04 | 0.00 | 0.04 | 458044 | 0.00 |
| 10 | ExtFP994 | Extend the fingerprinter with additional bits describing ring features | 0.02 | 0.58 | -0.56 | 195861 | 0.00 |
| 11 | GraphFP41 | Specialized version of the Fingerprinter which does not take bond orders into account | 0.02 | 0.32 | -0.30 | 309990.5 | 0.00 |
| 12 | GraphFP282 | Specialized version of the Fingerprinter which does not take bond orders into account | 0.01 | 0.56 | -0.55 | 194560.5 | 0.00 |
| 13 | GraphFP337 | Specialized version of the Fingerprinter which does not take bond orders into account | 0.01 | 0.25 | -0.24 | 334708.5 | 0.00 |
| 14 | GraphFP714 | Specialized version of the Fingerprinter which does not take bond orders into account | 0.00 | 0.19 | -0.19 | 357011 | 0.00 |
| 15 | GraphFP790 | Specialized version of the Fingerprinter which does not take bond orders into account | 1.00 | 0.99 | 0.01 | 443196 | 0.02 |
| 16 | GraphFP1011 | Specialized version of the Fingerprinter which does not take bond orders into account | 0.00 | 0.43 | -0.43 | 254711 | 0.00 |
| 17 | GraphFP1023 | Specialized version of the Fingerprinter which does not take bond orders into account | 0.01 | 0.57 | -0.56 | 194994 | 0.00 |
| 18 | PubchemFP35 | >= 4 S | 0.02 | 0.58 | -0.56 | 192586.5 | 0.00 |
| 19 | PubchemFP689 | O-C-C-C-C-C-C | 0.15 | 0.33 | -0.18 | 360602 | 0.00 |
| 20 | SubFPC36 | Presence of SMARTS Patterns for Functional Group Classification by Christian Laggner | 0.17 | 2.59 | -2.42 | 218651.5 | 0.00 |

* W (Wilcoxon test statistic) value is sum of the ranks in one of both groups, *NA: Unable to calculate the W-value.

**Table S4. The performance of models based on machine learning techniques developed using 2D and fingerprints of peptides.**

| **Machine Learning Technique**  **(Parameters)** | **Main Dataset** | | | | | **Validation Dataset** | | | | |
| --- | --- | --- | --- | --- | --- | --- | --- | --- | --- | --- |
|  | **Sen** | **Spc** | **Acc** | **MCC** | **AUROC** | **Sen** | **Spc** | **Acc** | **MCC** | **AUROC** |
| SVM full feature  (g=1e-05, c=1, j=2) | 23.25 | 96.51 | 59.59 | 0.29 | 0.70 | 25.26 | 94.62 | 59.57 | 0.28 | 0.75 |
| SVM after feature selection  (g=0.005, c=7, j=2) | 83.62 | 79.87 | 81.76 | 0.64 | 0.88 | 84.74 | 80.65 | 82.71 | 0.65 | 0.88 |
| Random Forest  (Ntree = 80) | 93.79 | 86.85 | 90.35 | 0.81 | 0.97 | 90.00 | 87.10 | 88.56 | 0.77 | 0.94 |
| SMO  (g=0.01, c=4) | 85.47 | 79.60 | 82.56 | 0.65 | 0.83 | 86.84 | 79.03 | 82.98 | 0.66 | 0.83 |
| J48  (c=0.3, m=1) | 91.94 | 85.77 | 88.88 | 0.78 | 0.91 | 86.32 | 86.56 | 86.44 | 0.73 | 0.91 |
| Naive Bayes  (Default) | 96.04 | 61.74 | 79.03 | 0.62 | 0.79 | 94.74 | 59.68 | 77.39 | 0.58 | 0.78 |

*** Sen:** Sensitivity, **Spc:** Specificity, **Acc:** Accuracy, **MCC:** Matthews Correlation Coefficient, **AUROC:** Area Under the Receiver Operating Characteristic curve.

**Table S5. The performance of SVM based models developed using binary profile of symbols obtained from terminals of SMILES format.**

| **Features**  **(Parameters)** | **Main Dataset** | | | | | **Validation Dataset** | | | | | |
| --- | --- | --- | --- | --- | --- | --- | --- | --- | --- | --- | --- |
|  | **Sen** | **Spc** | **Acc** | **MCC** | **AUROC** | **Sen** | **Spc** | **Acc** | **MCC** | **AUROC** |  |
| N25  (g=0.1, c=1, j=2) | 65.62 | 61.18 | 63.54 | 0.27 | 0.64 | 84.21 | 59.09 | 73.27 | 0.45 | 0.81 |  |
| N50  (g=0.01, c=9, j=1) | 73.55 | 73.43 | 73.49 | 0.47 | 0.79 | 71.97 | 70.63 | 71.32 | 0.43 | 0.81 |  |
| N100  (g=0.01, c=6, j=1) | 77.86 | 81.14 | 79.60 | 0.59 | 0.88 | 74.62 | 83.92 | 79.49 | 0.59 | 0.86 |  |
| C25  (g=0.005, c=2, j=2) | 67.27 | 62.76 | 64.71 | 0.30 | 0.67 | 70.18 | 62.30 | 66.10 | 0.33 | 0.72 |  |
| C50  (g=0.1, c=2, j=3) | 73.72 | 71.21 | 72.49 | 0.45 | 0.79 | 83.21 | 71.11 | 77.21 | 0.55 | 0.87 |  |
| C100  (g=0.05, c=1, j=2) | 79.81 | 76.92 | 78.27 | 0.57 | 0.87 | 83.97 | 82.24 | 83.04 | 0.66 | 0.89 |  |
| N25C25  (g=0.005, c=8, j=1) | 77.50 | 74.22 | 75.91 | 0.52 | 0.82 | 74.47 | 68.35 | 71.43 | 0.43 | 0.77 |  |
| N50C50  (g=0.01, c=6, j=2) | 84.92 | 83.16 | 84.05 | 0.68 | 0.91 | 78.48 | 81.21 | 79.88 | 0.60 | 0.89 |  |
| N100C100  (g=0.005, c=7, j=2) | 87.52 | 87.34 | 87.42 | 0.75 | 0.94 | 76.98 | 83.54 | 80.53 | 0.61 | 0.90 |  |

*** Sen:** Sensitivity, **Spc:** Specificity, **Acc:** Accuracy, **MCC:** Matthews Correlation Coefficient, **AUROC:** Area Under the Receiver Operating Characteristic curve, **N25/N50/N100:** First 25/50/100 elements from N-terminal, **C25/C50/C100:** First 25/50/100 elements from C-terminal, **N25C25/N50C50/N100C100:** First 25/50/100 elements from N-terminal as well as from C-terminal joined together.
